# New approaches to ranking countries for the allocation of development assistance for health: choices, indicators and implications

**DOI:** 10.1093/heapol/czx027

**Published:** 2018-02-05

**Authors:** Trygve Ottersen, Karen A Grépin, Klara Henderson, Crossley Beth Pinkstaff, Ole Frithjof Norheim, John-Arne Røttingen

**Affiliations:** 1 Department of International Public Health, Norwegian Institute of Public Health, Norway, Marcus Thranes gate 2, 0473 Oslo, Norway; 2 Oslo Group on Global Health Policy, Department of Community Medicine and Global Health and Centre for Global Health, University of Oslo, Kirkeveien 166, 0450 Oslo, Norway; 3 Department of Global Public Health and Primary Care, University of Bergen, Kalfarveien 31, 5018 Bergen, Norway; 4 Department of Health Sciences, Wilfrid Laurier University, Waterloo, 75 University Ave, W. Waterloo, ON, N2L3C5, Canada; 5 Independent Consultant, Warringah Street, North Balgowlah, NSW 2093, Australia; 6 Robert F. Wagner Graduate School of Public Service, New York University, 295 Lafayette St, New York, NY 10012, USA; 7 Department of Global Health and Population, Harvard T.H. Chan School of Public Health, Harvard University, 655 Huntington Ave, Boston, MA 02115, USA; 8 Department of Health Management and Health Economics, University of Oslo, Forskningsveien 3a/2b, 0373, Oslo, Norway; 9 Infectious Disease Control and Environmental Health, Norwegian Institute of Public Health, Lovisenberggata 8, 0456 Oslo, Norway

**Keywords:** Aid, development assistance for health, equity, health financing

## Abstract

The distributions of income and health within and across countries are changing. This challenges the way donors allocate development assistance for health (DAH) and particularly the role of gross national income per capita (GNIpc) in classifying countries to determine whether countries are eligible to receive assistance and how much they receive. Informed by a literature review and stakeholder consultations and interviews, we developed a stepwise approach to the design and assessment of country classification frameworks for the allocation of DAH, with emphasis on critical value choices. We devised 25 frameworks, all which combined GNIpc and at least one other indicator into an index. Indicators were selected and assessed based on relevance, salience, validity, consistency, and availability and timeliness, where relevance concerned the extent to which the indicator represented country’s health needs, domestic capacity, the expected impact of DAH, or equity. We assessed how the use of the different frameworks changed the rankings of low- and middle-income countries relative to a country’s ranking based on GNIpc alone. We found that stakeholders generally considered needs to be the most important concern to be captured by classification frameworks, followed by inequality, expected impact and domestic capacity. We further found that integrating a health-needs indicator with GNIpc makes a significant difference for many countries and country categories—and especially middle-income countries with high burden of unmet health needs—while the choice of specific indicator makes less difference. This together with assessments of relevance, salience, validity, consistency, and availability and timeliness suggest that donors have reasons to include a health-needs indicator in the initial classification of countries. It specifically suggests that life expectancy and disability-adjusted life year rate are indicators worth considering. Indicators related to other concerns may be mainly relevant at different stages of the decision-making process, require better data, or both.

Key Messages

- Stakeholders considered needs and health needs to be the most important concern to be captured by country classification frameworks for development assistance for health (DAH), followed by inequality, expected impact and domestic capacity.
- Integrating a health-needs indicator with gross national income per capita (GNIpc) in the classification framework makes a significant difference for many countries and country categories—and especially middle-income countries with high burden of unmet health needs—while the choice of specific indicator makes less of a difference.
- Donors have reasons to include one health-needs indicator in the initial classification of countries, and both life expectancy and disability-adjusted life year rate are indicators worth considering.
- For most donors, indicators related to other concerns—including equity—may be mainly relevant at different stages of the decision-making process, require better data, or both.

## Introduction

The distributions of income and health within and across countries are changing. Numerous countries have experienced impressive economic growth over the last two decades, and many have moved from low-income to middle-income status. A majority of the world’s poor and a majority of the world’s disease burden are now located in middle-income countries (MICs), which are often characterized by substantial inequalities in income, health and access to health services (Sumner 2012; Glassman *et al.* 2013; Røttingen *et al.* 2014). At the same time, there have been dramatic improvements in health outcomes and a shift in disease burden towards non-communicable diseases (Verguet *et al.* 2014; Norheim *et al.* 2015), while substantial inequalities in health both between and within countries remain (CSDH 2008; Salomon *et al.* 2012; WHO 2015). These transitions initially emerged alongside an unprecedented increase in development assistance for health (DAH), from $7 billion in 1990 to $34 billion in 2010 (2015 $US), but have more recently been accompanied by tepid growth (IHME 2016). Together, these changes have underscored challenges to donors’ current DAH allocation policies. In particular, most aid donors give gross national income per capita (GNIpc) a central role in classifying countries and allocating aid (cross-reference to article on current policies in this issue) (Ottersen *et al.* 2014). This role is now increasingly being questioned. One reason is that factors other than GNIpc are seen as relevant for countries’ capacity to address domestic health needs (Llavador and Roemer 2001; Tandon and Cashin 2010; Guillaumont 2011; Bárcena *et al.* 2012; Knack *et al.* 2012; Gupta and Mondal 2014; Resch *et al.* 2015). Another is that GNIpc is considered an inadequate reflection of countries’ level of unmet needs (Sen 1999; Stiglitz *et al.* 2010; Glennie 2011; Verbeke and Renard 2011; Sumner 2012; Glassman *et al.* 2013; R4D 2013; Ottersen *et al.* 2017a). It is also well known that GNIpc does not directly account for the distribution of income, health, and health services within countries (Ravallion 2001).

Together, this calls for new frameworks for classifying countries for the allocation of DAH; frameworks that go beyond GNIpc and incorporate a broader set of indicators. In response, the Equitable Access Initiative (EAI) was initiated in February 2015 to explore such frameworks. A group of researchers affiliated with the Norwegian Institute of Public Health (NIPH) was commissioned to provide input to the Initiative as one of four analytical teams. The specific objective of the NIPH team was to explore frameworks for classifying countries based on characteristics relevant to decisions on DAH. In the initial work, this was framed in terms of ‘external financing for health’, which includes traditional forms of DAH. The classifications were meant to guide donors in determining countries’ priority for DAH, which donors do both through decisions on what countries are eligible and through decisions on how much assistance each eligible country should be offered. This article presents the methods developed and employed by the NIPH team, reports and discusses the findings, and lays out recommendations for donors.

## Methods

### Stepwise approach to the design and assessment of frameworks

We developed and used a stepwise approach to the design and assessment of frameworks. This was done alongside a series of interviews and consultations with stakeholders, which we further discuss below. These various activities were pursued in parallel and informed each other.

The stepwise approach to the design and assessment of frameworks comprised four steps: delineation of normative basis; screening of indicators; design of candidate frameworks; and assessment of frameworks. The approach was developed on the basis of a review of previous efforts of framework and index construction in various fields and with the aim of exposing the key choices involved in the design and assessment of classification frameworks.

#### Normative basis

Decisions concerning DAH are inherently based on value choices, but these are often left implicit or poorly defined. We sought to clearly delineate a normative basis for the allocation of DAH by identifying and defining the basic, underlying country-level concerns that motivate the allocation of aid by donors among partner countries. To this end, we reviewed the literature on health financing, development assistance and distributional justice, and we elicited stakeholders’ views through consultations and interviews (Llavador and Roemer 2001; Bell and Fink 2005; Cogneau and Naudet 2007; Anderson 2008; Guillaumont 2008; Ottersen *et al.* 2014; Røttingen *et al.* 2014). Through our investigations, we characterized motivations into 3 + 1 general concerns: health needs, domestic capacity, expected impact, and the cross-cutting concern (+1) for equity (Table 1). These are meant to be concerns that all or nearly all stakeholders find relevant for the allocation of aid across countries. At the same time, we found that stakeholders disagreed on the relative importance of these concerns and on how to operationalize them, including which indicators best capture each concern.

**Table 1.** Definitions of the basic concerns

*Health needs*: A population’s need for improvement in health or the determinants of health, including health-service coverage
*Domestic capacity*: The capacity of countries to address domestic needs without DAH^a^
*Expected impact*: The expected impact of DAH in terms of changes in health or the determinants of health, including health-service coverage
*Equity* (*cross-cutting*): The fair distribution of resources, services, and outcomes across individuals, groups and populations^b^

a Capacity can include ability to pay and fiscal space, but also go beyond merely financial factors.

b Equity is a cross-cutting concern sensitive to how well the three other concerns are addressed.

#### Indicators

We identified candidate country-level indicators for classification frameworks by first crafting a list of potential indicators (see Supplementary File S1). This list was based on a number of review articles, our own literature review and the list of candidate indicators generated at the EAI Expert Panel meeting in February 2015 (Anderson 2008; Salomon *et al.* 2012; Basu *et al.* 2014; Ottersen *et al.* 2014; Norheim *et al.* 2015; Vázquez and Sumner 2015).

We then proceeded to screen the indicators on the basis of relevance, salience, validity, consistency, and availability and timeliness (Bonita *et al.* 2006; OECD 2008; UNSD 2015). Relevance was defined as the extent to which the indicator provides meaningful information about country characteristics related to health needs, domestic capacity, expected impact, or equity—as these four concerns are defined in Table 1. ‘Meaningful’ was here judged relative to the specific purpose of classifying countries for a wide range of actors and in a wide range of settings. Salience was defined as the extent to which the indicator can be easily understood by policy makers and the general public. Validity was defined as the extent to which the indicator measures what it purports to measure, while consistency was understood as the degree to which data measurements are stable when repeated if the situation remains unchanged. Availability was seen to depend on the number of missing values across countries, while timeliness depended on the recency and expected regularity of updates.

#### Design

We designed 25 candidate frameworks to allow for broad comparison and analysis of key value choices. We sought simple frameworks in the form of indices that were easy to understand for stakeholders, and we sought frameworks that together covered a wide range of indicators and all the basic concerns.

The design of each framework followed the same four steps. Our starting point for each index was GNIpc. This was motivated by the view that while GNIpc alone is insufficient for classifying countries, national income per capita is likely to be an important part of any classification framework, at least in the short to medium term. The GNIpc estimates used were not adjusted for purchasing power parity (PPP), which is in line with the World Bank classification and most eligibility thresholds and allocation policies employed today. Beyond GNIpc, most of the indices we constructed included only one additional indicator. Each indicator was chosen from the long list of indicators based on the screening criteria and the goal of comprehensiveness for the final set of candidate frameworks. A detailed description, including data sources, of the indicators integrated in one or more frameworks are provided in Appendix 1. We next specified the prioritization rule—a step that is often left implicit. The prioritization rule specifies what happens to a country’s priority for DAH when the country’s value of a given indicator increases (Ottersen *et al.* 2017b). For example, according to a common rule for GNIpc, rank and priority decrease monotonically with increases in GNIpc. The third sub-step was min–max normalization (OECD 2008). This gives the indicators an identical range [0, 1] and facilitates comparison by subtracting from each observation the lowest observed value and dividing by the range of observed indicator values.

The fourth step was weighting and aggregation. When combining two or more indicators into a composite index, one needs to determine their relative weight (OECD 2008). We used equal weighting as the starting point throughout, that is, all indicators in each framework were assigned the same weight. Although equal weighting by no means is value neutral, it facilitates intuitive understanding of the frameworks and their implications. For one framework, we applied weights informed by the online survey (cross-reference to article on discrete choice experiment by Grépin *et al.* in this issue). Finally, to aggregate two or more products of a weight and a normalized indicator into a composite index, we employed linear aggregation, that is, straightforward summation of these products (OECD 2008).

#### Assessment

We assessed candidate frameworks through a direct comparison of their implications in terms of the ranking of countries, where higher ranking meant higher priority for DAH. Specifically, we compared the ranking of individual countries as determined by each of the 25 frameworks relative to its ranking based on GNIpc alone. These changes in rank indicate *which* countries and country categories are most likely to be affected and *how much* each is likely to be affected—in terms of eligibility or allocated amounts—if one moves from GNIpc to a broader framework. Such basic understanding of the implications of different frameworks—alongside more direct considerations of the principles and criteria involved—is helpful for donors when they assess or revise the frameworks they currently use. Such an understanding is also useful for other stakeholders and the broader community when debating how countries are best classified for guiding the allocation of DAH.

Our initial sample of countries included all countries classified as low-income countries (LICs) and middle-income countries (MICs) for which GNIpc was available (*N* = 131). Although rankings were generated for all these countries, we concentrated on five focus countries for illustrative purposes: Ethiopia, India, Mali, Nigeria and South Africa. This set was chosen to jointly cover different levels of income (including LICs, lower–middle-income countries (LMICs) and upper–middle-income countries (UMICs)), health expenditures, health service coverage, health outcomes, and inequalities in income and health. This set of countries should thus help demonstrate key features of classification frameworks. Table 2 exhibits central characteristics of the focus countries and underscores how the countries differ in multiple dimensions.

**Table 2.** Central characteristics of focus countries

Country	Income class	GNIpc	LE	DALYR	HIVR	Debt	GHEpc	ILE	Gini	SBA
Ethiopia	LIC	470	64	48 475	1.2	1.4	15	30	34	23
India	LMIC	1530	67	39 494	0.1	2.1	20	25	34	67
Mali	LIC	830	58	86 628	1.3	0.8	8	29	33	40
Nigeria	LMIC	2700	53	73 320	3.3	0.1	32	41	43	38
South Africa	UMIC	7410	57	55 894	18.8	2.5	287	26	65	95

Income class, World Bank income class for the fiscal year 2016; GNIpc, gross national income per capita (Atlas, current $US); DALYR, disability-adjusted life year rate (per 100 000 individuals); HIVR, HIV prevalence rate (% of population aged 15–49); Debt, total debt service (% of GNI); GHEpc, government health expenditure per capita (current $US); ILE, inequality in life expectancy; Gini, Gini index for income; SBA, skilled birth attendance rate (% of total deliveries). Data sources and timing as described in Appendix 1, except for SBA for South Africa (year 2008).

Implications for five categories of countries were also examined. Three of these are World Bank income classes—LICs, LMICs and UMICs—for the fiscal year 2016. The other two are the 20% of countries with the lowest life expectancy and the 20% of countries with the lowest ratio of GNI to disability-adjusted life years (DALYs). While focus on the bottom quintile is common, the choice of a 20% threshold is of course somewhat arbitrary. The countries affiliated with each category are listed in Appendix 2. Implications across income classes were examined because much of the debate is framed in terms of the role of MICs compared with that of LICs. Similarly, implications for the two other country categories were examined because concerns for countries with profound health needs and the relationship between economic capacity and disease burden figure prominently in the current debate. Yet, it is worth noting that 96% of the 20% of countries with the lowest GNI-DALY ratio in our sample are classified as LICs. The relationship between this ratio and income class has been examined in more detail elsewhere, including in the specific context of AIDS (Resch *et al.* 2015).

In addition to direct comparisons of rankings, we calculated Spearman coefficients for the rank correlation between GNIpc and each of the frameworks. These coefficients indicate the overall number and magnitude of rank changes that come with the various frameworks. A lower rank correlation between GNIpc and the country scores following from a framework suggests that moving from GNIpc alone to that framework in question is more likely to affect the overall ranking of countries. Although we were primarily after comparing GNIpc with frameworks, we also calculated Spearman coefficients for the rank correlation between GNIpc and each of the bare indicators.

For indicators with only a few years of missing data by country, we employed linear interpolation to impute missing values for the preferred year, using available data for the years 2000–13. In addition, for each category of frameworks—health-needs, capacity, impact, disease-specific, other two-criterion and multi-criterion frameworks—only countries with available data (after imputation) for all the indicators pertaining to the framework category were included. This strategy was pursued to reduce the effect of missing values countries’ change in rank.

### Elicitation of stakeholder views

We elicited the views of stakeholders through consultations, interviews and an online survey. Findings from the online survey is reported by Grépin et al in this issue. Six consultations were organized by the EAI in Geneva or New York. Here, we presented preliminary plans, methods, and findings and received input from a wide range of stakeholders. These represented the EAI Convening Partners, multi- and bilateral donors, partner countries, civil society organizations, the private sector and academia.

To gather further input, we conducted twenty semi-structured interviews with stakeholders in August and September 2015. Interviewees were identified through a screening of members of boards and committees at the Global Fund to Fight AIDS, Tuberculosis and Malaria and at Gavi, members of the EAI Technical Working Group, members of the EAI Expert Panel and experts known to the team or with published work in the field. We selected the individuals to invite for an interview on the basis of their expertise on topics central to this study and their contribution to diversity in the overall sample of interviewees. Nineteen of the interviews took place over the phone, and one was conducted face-to-face. Nine of the 20 interviewees were members of either the EAI Conveners’ Technical Working Group or the EAI Expert Panel. The stakeholders interviewed represented academic institutions (six), civil society or non-governmental organizations (four), multilateral organizations (four), bilateral donors (three), partner-country governments (two) and a philanthropic foundation (one), with several stakeholders representing more than one institution. Partner country stakeholders represented various institutions in India, Pakistan, South Africa, Thailand and Zimbabwe. The interviewees held senior positions within their institutions, most with responsibility for policy, global health, access, human development or a combination.

The interview questions were developed in dialogue with the EAI. The interviews concentrated on stakeholder views on classification frameworks and characteristics relevant to decisions on DAH. Questions pertained to the purpose of such frameworks, the role and relevance of four concerns (needs and health needs, capacity, impact and inequality) and indicators.

## Results

Primary findings were of four kinds: stakeholder views, menu of candidate frameworks, indicator evaluations and framework implications. In addition, the stepwise approach developed can be useful for donors and stakeholders in their design or assessment of frameworks.

### Stakeholder views

Stakeholders reported views pertaining to the role of frameworks, relevant concerns, and relevant indicators.

#### Role of frameworks

Respondents considered frameworks for classifying countries to be potentially useful as an *initial* guide to decisions on DAH. This included decisions on eligibility, terms of contributions, and the size of allocations. Many respondents asserted that one also needs a framework for choosing the modality of assistance, e.g. whether to focus on financial assistance, technical assistance, capacity building, or advocacy. At the same time, most respondents emphasized that the role of classification frameworks in the overall decision making progress is circumscribed and that a wider set of factors needs to be considered after a classification framework has been applied. These included various qualitative factors, for example related to a country’s economic and health policies, social issues, tax systems and performance; respondents perceived as less apt for being captured by a framework.

Respondents generally emphasized that the frameworks needed to be simple and transparent. At the same time, several respondents noted the risk of frameworks being oversimplified and insufficiently sensitive to the complexities in the allocation of DAH.

#### Relevant concerns

Respondents were directly asked about four areas of concern: needs and health needs, capacity, expected impact and inequality. Of these, needs and health needs was identified as the most important by stakeholders, followed by inequality. Several respondents asserted that we must avoid ‘double penalty’, where neither the national government nor external donors accepts responsibility for poor people with unmet health needs.

Several stakeholders suggested that considerations of capacity and expected impact are important, but often in ways external to the initial classification of countries and thus to the classification frameworks. For example, it was suggested that capacity and expected impact help address questions about *how* to meet needs. This included questions about time horizon and modality of assistance. Capacity was generally considered the least important of the four concerns, and many emphasized aspects of capacity that they perceived to be different from ‘ability to address health needs without external financing.’ For example, several saw capacity as primarily linked to the strength of the country’s health system and infrastructure, independently of the country’s need for external assistance.

Finally, several stakeholders made a distinction between funding by bilaterals and multilaterals and expressed that the former have more flexibility but are also more subject to political verities and geopolitical considerations.

#### Relevant indicators

Respondents deemed GNIpc to be relevant, but emphasized the need to go beyond this and consider additional factors. The statement by one interviewee that GNIpc is the ‘least worst indicator’ represented a view shared by many respondents. The interviewees noted that GNIpc is updated every year, available for all countries, easily understood, correlated with other indicators and transparent.

Although most acknowledged the role of GNIpc as a principal indicator, there were caveats and concerns. A number of these were related to the classification of MICs. Here, stakeholders noted the need for sensitivity to the following issues: dramatic and rapid shifts in GNIpc levels that may prematurely suggest greater capacity; the limitations of GNIpc in identifying the extensive health needs and poverty in MICs (subnational, at-risk groups); and the importance of broader indicators and criteria designed to incentivize domestic investment in health (rather than displacing domestic spending) or identify gaps in coverage and capacity. More generally, and in line with their ranking of concerns, respondents identified measures of health needs and inequalities as the most important indicators to include in a classification framework alongside GNIpc.

### Menu of frameworks

Based on the screening of indicators, 25 candidate frameworks were generated, as shown in Table 3. Except for the two multi-criterion frameworks, every framework integrates GNIpc and one other indicator into an index. The name of each framework was given by the indicator integrated with GNIpc. Table 3 also shows the prioritization rule used for each indicator, i.e. what happens to a country’s priority for DAH when the country’s value of a given indicator increases. According to the prioritization rule used for GNIpc, a country’s priority decreases with GNIpc.

**Table 3.** Menu of frameworks

Framework and indicator supplementing GNIpc	Abbreviated name	Prioritization rule	Mean	Standard deviation	Min	Max
*Health-needs frameworks*						
Under-five mortality rate (per 1000 live births)	U5MR	+	45	35	5	167
Under-sixty mortality rate (per 1000 adults)	U60MR	+	212	111	56	715
Life expectancy (years)	LE	−	67	8	46	80
Healthy life expectancy (years)	HALE	−	59	6	42	70
Disability-adjusted life years rate (per 100 000 individuals)	DALYR	+	40 043	17 067	17 647	87 948
Age-standardised disability-adjusted life years rate (per 100 000 individuals)	DALYR_AS	+	43 017	16 521	21 541	99 360
*Capacity frameworks*						
Debt service (% of GNI)	Debt	+	4	5	−3	28
Tax ratio (% of GDP)	Tax	−	16	13	−44	81
Total health expenditure per capita (current $US)	THEpc	−	252	231	13	1085
Government health expenditure per capita (current $US)	GHEpc	−	154	163	6	776
*Impact frameworks*						
Absolute improvement in under-five mortality rate (per 1000 live births)	cU5MR	+	24	22	−6	92
Relative improvement in under-five mortality rate (%)	rcU5MR	+	32	15	−39	67
Absolute improvement in skilled birth attendance rate (per 1000 live births)	cSBA	+	10	14	−38	59
Relative improvement in skilled birth attendance rate (%)	rcSBA	+	36	100	−43	1000
*Disase-specific frameworks*						
Maternal mortality ratio (per 1000 live births)	MMR	+	241	262	4	1460
HIV prevalence rate (% of population aged 15–49)	HIVR	+	2	5	0	28
Tuberculosis prevalence rate (per 100 000 population)	TBR	+	205	203	7	945
*Other two-criterion frameworks*						
Inequality in life expectancy	ILE	+	22	12	5	51
Gini index for income	Gini	+	40	9	25	65
Income share held by bottom 40% (% of total income)	Income40	−	17	5	6	29
Skilled birth attendance rate (% of total deliveries)	SBA	−	82	24	19	139
Coverage of three doses of vaccine against diphtheria, tetanus, and pertussis (% of children aged 12–23 months)	DTP3	−	87	13	23	99
Out-of-pocket payments (% of total health expenditure)	OOPP	+	36	18	0	76
*Multi-criterion frameworks*						
LE, ILE and SBA with equal weights	MCF_EQ	*	*	*	*	*
LE, ILE and SBA with weights informed by online survey	MCF_SU	*	*	*	*	*

+ implies that a country’s priority for DAH increases when the country’s value of the indicator increases. – implies that a country’s priority for DAH increases when the country’s value of the indicator decreases. * indicates that a combination of the values provided above is what is relevant. Further information about the indicators is provided in Appendix 1.

As exhibited in Table 3, the stepwise approach generated six general health-needs frameworks, four domestic-capacity frameworks, four impact frameworks, three disease-specific frameworks, six other two-criterion frameworks which cut across one or more of the basic concerns and two multi-criterion frameworks integrating four indicators: GNIpc, life expectancy, inequality in life expectancy and skilled birth attendance rate (SBA). One of the multi-criterion frameworks (MCF_EQ) utilizes equal weights, while the other (MCF_SU) utilizes weights informed by the online survey (cross-reference to article by Grépin *et al.* in this issue). Specifically, we assigned a weight of 0.1 to GNIpc, 0.3 to life expectancy, 0.4 to inequality in life expectancy and 0.2 to SBA.

### Indicator evaluations

We found that the indicators integrated in one or more frameworks varied considerably in terms of relevance, salience, validity, consistency, and availability and timeliness. The health-needs indicators generally scored well against relevance, although the applicability of under-five mortality rate (U5MR) and under-60 mortality rate (U60MR) may be restricted by them only capturing mortality before the age of 5 and between the ages of 15 and 60, respectively. In contrast, life expectancy, healthy life expectancy (HALE), disability-adjusted life year rate (DALYR) and age-standardized disability-adjusted life year rate (DALYR_AS) are sensitive to all deaths, at all ages, and from all causes. At the same time, the latter three of these differed from the other indicators by integrating morbidity in addition to mortality. The gap measures DALYR and DALYR_AS also stood out by facilitating decomposition of health needs with regard to cause, risk factor and age structure (Murray *et al.* 2012). The health-needs’ indicators generally scored well also against salience, validity, consistency, and availability and timeliness, with U5MR and life expectancy being the top performers, as estimates are provided by several reliable institutions, available for nearly all countries and frequently updated.

While domestic capacity was found to be a relevant concern, the capacity indicators (beyond GNIpc) generally scored less well against relevance. A key reason was that the relationship between each of the indicators and rank in a classification framework appeared more convoluted than for the health-needs indicators. We also found that it is tricky to draw the line between government choice and external circumstances (Llavador and Roemer 2001; Cogneau and Naudet 2007) and that the question about what kind of capacity is most relevant for the allocation of DAH is still very open. For example, a high level of government health expenditure per capita (GHEpc) represents a more downstream and health-specific type of capacity than a high level of general government revenue or a low level of external debt. At the same time, the capacity indicators scored overall relatively well against salience, validity, consistency, and availability and timeliness. Estimates on total health expenditure per capita (THEpc) and GHEpc were most readily available, but comes with important concerns about the data quality, especially for LICs.

The impact indicators scored relatively well against validity, consistency, and availability and timeliness, with the U5MR-related indicators performing somewhat better than those linked to SBA. Relevance for a classification framework was found to be the main challenge for these impact indicators, even for donors focusing on child or maternal health. While expected impact was found to be a relevant concern, the link between the impact indicators and rank in a classification framework appeared far from straightforward (Buiter 2007; Pearson 2011). One reason is that improvements in the recent past may have little or nothing to do with development assistance. Another reason is that even it did so in the past, the future may be quite different. The context may have changed, or the indicator for which improvement is sought may have reached a level at which marginal changes are more difficult to achieve.

The disease-specific indicators were found to be too narrow for donors with health mandates broader than HIV, tuberculosis, or maternal mortality. Overall, these indicators scored moderately well against validity, consistency, and availability and timeliness.

The category of other indicators is a heterogeneous group. This was also reflected in their evaluation. Inequality was generally found relevant for the allocation of DAH. However, the relevance of inequality indicators for the classification of countries was challenged by the complex relationship between inequality levels and priority for DAH. Greater inequality may indicate higher needs and suggest higher priority, but greater inequality may also indicate higher capacity to address needs—including through redistribution—and thus suggest lower priority (Ceriani and Verme 2013). In addition, if greater inequalities implies more DAH, countries have less incentive to reduce these (Basu *et al.* 2014). The inequality indicators were also found to have limitations with regard to validity, consistency, and availability and timeliness. Both the Gini and Income40 are updated only irregularly and available only for a limited number of countries for any given year. Inequality in life expectancy is updated annually but has been tested and scrutinized only to a limited extent.

The final three indicators—SBA; third dose of vaccine against diphtheria, tetanus, pertussis (DTP3); and out-of-pocket payments (OOPPs)—were found to partly signal need. However, the relevance of SBA and DTP3 in a classification framework was also found to be challenged by their restricted focus, which may be too narrow for many donors. All three indicators were found to be linked to data of quite low quality. 

### Framework implications

The Spearman coefficients for all the 25 frameworks are shown in Table 4, alongside the Spearman coefficients for the correlation between GNIpc and each bare indicator. The high coefficients for the frameworks are partly due to the fact that GNIpc itself is part of these.

**Table 4.** Spearman coefficients for correlation between frameworks and between indicators

Framework and indicator supplementing GNIpc	Abbreviated name	Spearman rank correlation (framework)	Spearman rank correlation (indicator)
*Health-needs frameworks*			
Under-five mortality rate	U5MR	0.93	0.76
Under-sixty mortality rate	U60MR	0.89	0.61
Life expectancy	LE	0.90	0.70
Healthy life expectancy	HALE	0.93	0.70
Disability-adjusted life years rate	DALYR	0.92	0.66
Age-standardised disability-adjusted life years rate	DALYR_AS	0.93	0.70
*Capacity frameworks*			
Debt service	Debt	0.73	−0.54
Tax ratio	Tax	0.97	0.37
Total health expenditure per capita	THEpc	0.99	0.95
Government health expenditure per capita	GHEpc	0.99	0.93
*Impact frameworks*			
Absolute improvement in under-five mortality rate	cU5MR	0.94	0.69
Relative improvement in under-five mortality rate	rcU5MR	0.90	0.16
Absolute improvement in skilled birth attendance rate	cSBA	0.91	0.41
Relative improvement in skilled birth attendance rate	rcSBA	0.98	0.49
*Disase-specific frameworks*			
Maternal mortality ratio	MMR	0.96	0.76
HIV prevalence rate	HIVR	0.86	0.41
Tuberculosis prevalence rate	TBR	0.87	0.49
*Other two-criterion frameworks*			
Inequality in life expectancy	ILE	0.93	0.75
Gini index for income	Gini	0.75	−0.06
Income share held by bottom 40%	Income40	0.76	−0.10
Skilled birth attendance rate	SBA	0.93	0.68
Coverage of three doses of vaccine against diphtheria, tetanus, and pertussis	DTP3	0.93	0.44
Out-of-pocket payments	OOPP	0.79	0.27
*Multi-criterion frameworks*			
LE, ILE, and SBA with equal weights	MCF_EQ	0.97	
LE, ILE, and SBA with weights informed by online survey	MCF_SU	0.95

**Appendix 1.** Indicators included in one or more frameworks

Indicator	Brief description	Long description	Year	Source	World Development Indicator code	Countries with missing data (%)^a^	
GNIpc	Gross national income per capita, Atlas method (current US$)	The gross national income, converted to U.S. dollars using the World Bank Atlas method, divided by the midyear population	2013	World Bank	NY.GNP.PCAP.CD	0	
U5MR	Under-five mortality rate (per 1000 live births)	The probability per 1000 that a newborn baby will die before reaching age five, if subject to age-specific mortality rates of the specified year	2013	UN Inter-agency Group for Child Mortality Estimation	SH.DYN.MORT	1	
U60MR	Mortality rate between ages 15 and 60 (per 1000 adults)	The probability of dying between the ages of 15 and 60—that is, the probability of a 15-year-old dying before reaching age 60, if subject to current age-specific mortality rates between those ages	2013	United Nations Population Division	SP.DYN.AMRT.FE and SP.DYN.AMRT.MA	4	
LE	Life expectancy at birth (years)	The number of years a newborn infant would live if prevailing patterns of mortality at the time of its birth were to stay the same throughout its life	2013	World Bank	SP.DYN.LE00.IN	2	
HALE	Healthy life expectancy at birth (healthy life years)	The number of years a newborn infant would live if prevailing patterns of mortality at the time of its birth were to stay the same throughout its life, adjusted for the expected disability in those years	2013	IHME	N/A	2	
DALYR	Disability-adjusted life year rate (per 100 000 individuals)	The number of years of life lost due to premature mortality (YLLs) and years lived with disability (YLDs) per 100 000 population	2013	IHME	N/A	2	
DALYR_AS	Age-standardised disability-adjusted life year rate (per 100 000 individuals)	The number of years of life lost due to premature mortality (YLLs) and years lived with disability (YLDs) per 100 000 population, standardized by age	2013	IHME	N/A	2	
MMR	Maternal mortality ratio (per 100 000 live births)	The number of women who die from pregnancy-related causes while pregnant or within 42 days of pregnancy termination per 100 000 live births	2013	WHO	SH.STA.MMRT	4	
HIVR	Prevalence of HIV (% of population ages 15-49)	The percentage of people aged 15–49 who are infected with HIV	2013	UNAIDS	SH.DYN.AIDS.ZS	0	
TBR	Tuberculosis prevalence rate (per 100 000 population)	The estimated number of TB cases (all forms) at a given point in time, expressed as the rate per 100 000 population	2013	WHO	N/A	1	
Debt	Total debt service (% of GNI)	The sum of principal repayments and interest actually paid in currency, goods, or services on long-term debt, interest paid on short-term debt, and repayments (repurchases and charges) to the IMF	2013	World Bank	DT.TDS.DECT.GN.ZS	10	
Tax	Tax revenue (% of GDP)	Compulsory transfers to the central government for public purposes. Certain compulsory transfers such as fines, penalties, and most social security contributions are excluded. Refunds and corrections of erroneously collected tax revenue are treated as negative revenue	2013	IMF	GC.TAX.TOTL.GD.ZS	21	
THEpc	Total health expenditure per capita (current US$)	Sum of public and private health expenditures as a ratio of total population. It covers the provision of health services (preventive and curative), family planning activities, nutrition activities, and emergency aid designated for health, but does not include provision of water and sanitation	2013	WHO	SH.XPD.PCAP		2
GHEpc	Government health expenditure per capita (current US$)	Product of total health expenditure per capita (current US$) and public health expenditure (% of total health expenditure)	2013	WHO	SH.XPD.PCAP and SH.XPD.PUBL		2
cU5MR	Absolute change in under-five mortality rate, 2003–13	Absolute change in under-five mortality rate between 2003 and 2013, using 2003 as the base year	2003–13	UN Inter-agency Group for Child Mortality Estimation	SH.DYN.MORT (as input)		1
rcU5MR	Relative change in under-five mortality rate, 2003–13	Relative change in under-five mortality rate between 2003 and 2013, using 2003 as the base year.	2003–13	UN Inter-agency Group for Child Mortality Estimation	SH.DYN.MORT (as input)		1
cSBA	Absolute change in skilled birth attendance rate, 2003–2013	Absolute change in skilled birth attendance rate between 2003 and 2013, using 2003 as the base year	2003–13	UNICEF	SH.STA.BRTC.ZS (as input)		4
rcSBA	Relative change in skilled birth attendance rate, 2003–13	Relative change in skilled birth attendance rate between 2003 and 2013, using 2003 as the base year	2003–13	UNICEF	SH.STA.BRTC.ZS (as input)		4
ILE	Inequality in life expectancy	Inequality in distribution of expected length of life based on data from life tables estimated using the Atkinson inequality index	2013	UNDP	N/A		5
GINI	GINI index	The extent to which the distribution of income (or, in some cases, consumption expenditure) among individuals or households within an economy deviates from a perfectly equal distribution	Most recent (2003–2013)	World Bank	SI.POV.GINI		22
Income40	Income share held by bottom 40% of population (% of total income)	Percentage share of total income or consumption that accrues to the 40% of the population with the lowest income or consumption	2013	World Bank	SI.DST.02ND.20 and SI.DST.FRST.20		29
SBA	Births attended by skilled health staff (% of total deliveries)	The percentage of deliveries attended by personnel trained to give the necessary supervision, care, and advice to women during pregnancy, labor, and the postpartum period; to conduct deliveries on their own; and to care for newborns	2013	UNICEF	SH.STA.BRTC.ZS		4
DTP3	DTP3 immunization coverage (% of children ages 12–23 months)	The percentage of children ages 12–23 months who received vaccinations before 12 months or at any time before the survey. A child is considered adequately immunized against diphtheria, pertussis (or whooping cough), and tetanus (DPT) after receiving three doses of vaccine	2013	WHO and UNICEF	SH.IMM.IDPT		2
OOPP	Out-of-pocket health expenditure (% of total expenditure on health)	The percentage of total health expenditure that is direct outlays by households, including gratuities and in-kind payments, to health practitioners and suppliers of pharmaceuticals, therapeutic appliances, and other goods and services whose primary intent is to contribute to the restoration or enhancement of the health status of individuals or population groups. It is a part of private health expenditure	2013	WHO	SH.XPD.OOPC.TO.ZS		2

a This is the share of countries with missing data for the given indicator among the countries included in our study sample.

**Appendix 2.** Countries included in each country category

Low-income countries	Lower–middle-income countries	Upper–middle-income countries
Afghanistan^b^	Armenia	Albania
Benin^b^	Bangladesh	Algeria
Burkina Faso^a^^,b^	Bhutan	Angola^a^
Burundi^a^^,b^	Bolivia	Azerbaijan
Cambodia	Cabo Verde	Belarus
Central African Republic^a^^,b^	Cameroon^a^	Belize
Chad^a^^,b^	Congo, Rep.^a^	Bosnia and Herzegovina
Comoros	Cote d'Ivoire^a^	Botswana^a^
Congo, Dem. Rep.^a^^,b^	Djibouti	Brazil
Eritrea^b^	Egypt, Arab Rep.	Bulgaria
Ethiopia^b^	El Salvador	China
Gambia, The^a^^,b^	Georgia	Colombia
Guinea^a,b^	Ghana	Costa Rica
Guinea-Bissau^a^^,b^	Guatemala	Cuba
Haiti	Guyana	Dominica
Liberia^b^	Honduras	Dominican Republic
Madagascar^b^	India	Ecuador
Malawi^a^^,b^	Indonesia	Fiji
Mali^a^^,b^	Kenya	Gabon
Mozambique^a^^,b^	Kiribati	Grenada
Nepal	Kosovo	Iran, Islamic Rep.
Niger^a^^,b^	Kyrgyz Republic	Iraq
Rwanda^b^	Lao PDR	Jamaica
Sierra Leone^a^^,b^	Lesotho^a^^,b^	Jordan
South Sudan^a^^,b^	Mauritania	Kazakhstan
Tanzania^b^	Micronesia, Fed. Sts.	Lebanon
Togo^a^^,b^	Moldova	Libya
Uganda^a,b^	Morocco	Macedonia, FYR
Zimbabwe^b^	Nicaragua	Malaysia
	Nigeria^a^	Maldives
	Pakistan	Marshall Islands
	Papua New Guinea	Mauritius
	Philippines	Mexico
	Samoa	Mongolia
	Sao Tome and Principe	Montenegro
	Senegal	Namibia
	Solomon Islands	Palau
	Sri Lanka	Panama
	Sudan	Paraguay
	Swaziland^a^	Peru
	Syrian Arab Republic	Romania
	Tajikistan	Serbia
	Timor-Leste	South Africa^a^
	Ukraine	St. Lucia
	Uzbekistan	St. Vincent and the Grenadines
	Vanuatu	Suriname
	Vietnam	Thailand
	West Bank and Gaza	Tonga
	Yemen, Rep.	Tunisia
	Zambia^a^	Turkey
		Turkmenistan
		Tuvalu

Income classes for fiscal year 2016 based on gross national income per capita (GNIpc) for 2014: Low-income countries (GNIpc ≤ $1045); lower-middle-income countries (GNIpc $1046–$4125); upper-middle-income countries (GNIpc $4126–$12 735). Source: http://siteresources.worldbank.org/DATASTATISTICS/Resources/OGHIST.xls.

a Among the 20% of countries with the lowest life expectancy.

b Among the 20% of countries with the lowest ratio of GNI to disability-adjusted life years (DALYs).

#### Health-needs frameworks

We found that integrating a health-needs indicator with GNIpc made a substantial difference for many countries and country categories, while the choice of health-needs indicator made less of a difference.

As for general trends, the Spearman coefficients for correlations between GNIpc and each of the health-needs frameworks were all fairly high, ranging from 0.89 (U60MR), via 0.90 (life expectancy (LE)) and 0.92 (DALYR), to 0.93 (U5MR, HALE and DALYR_AS). Correlations between GNIpc and each of the health-needs indicators were obviously lower, as shown in Table 4.

**Figure 1.**
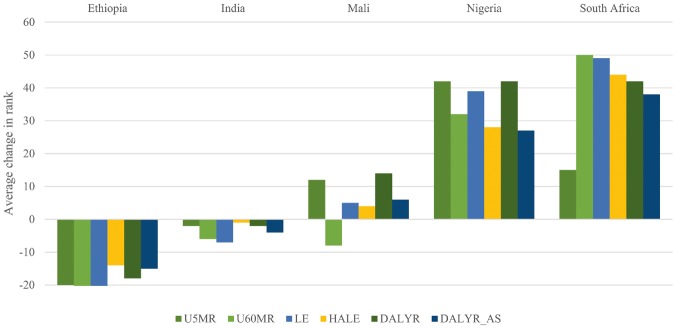
Changes in rank when moving from GNIpc alone to health-need frameworks (focus countries). U5MR, framework integrating under-five mortality rate; U60MR, framework integrating under-sixty mortality rate (ages 15–60); LE, framework integrating life expectancy; HALE, framework integrating healthy life expectancy; DALYR, framework integrating disability-adjusted life year rate; DALYR_AS, framework integrating age-standardized disability-adjusted life year rate

While correlations between GNIpc and each of the health-needs frameworks were fairly high, the departures from perfect correlation still allow for marked changes in rank for individual countries. Figure 1 shows how the rank of the five focus countries changed as one moved from GNIpc alone to each of the health-needs frameworks, where higher rank implies higher priority for DAH. Changes for all countries are presented in Supplementary File S2.

Nigeria and South Africa got a substantially higher rank and thus priority, whereas Ethiopia got a significantly lower rank. For India and Mali, the changes are less pronounced or more mixed. The figure also demonstrates that the differences across health-needs frameworks for the most part were quite small.

**Figure 2.**
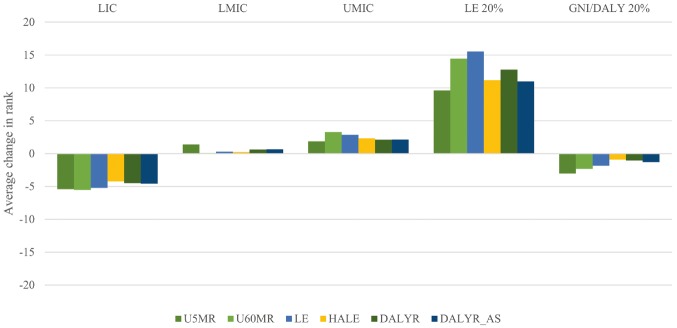
Average changes in rank for countries when moving from GNIpc alone to health-need frameworks (country categories). U5MR, framework integrating under-five mortality rate; U60MR, framework integrating under-sixty mortality rate (ages 15–60); LE, framework integrating life expectancy; HALE, framework integrating healthy life expectancy; DALYR, framework integrating disability-adjusted life year rate; DALYR_AS, framework integrating age-standardized disability-adjusted life year rate; LIC, low-income countries; LMIC, lower-middle-income countries; UMIC, upper-middle-income countries; LE 20%, 20% of countries with the lowest life expectancy; GNI/DALY 20%, 20% of countries with lowest ratio of GNI to DALYs

With regard to the categories of countries, Figure 2 shows the average changes in country rank as one moved from GNIpc alone to health-needs frameworks.

On an average, LICs got a somewhat lower rank when moving from GNIpc alone, irrespective of the health needs framework. Conversely, the LMIC and UMIC groups got a higher rank from any move away from GNIpc alone, but this increase was small. The countries with the lowest life expectancies experienced a quite substantial increase in rank across all health-needs frameworks. For the countries with the lowest GNI-DALY ratios, however, the inclusion of a health-needs indicator led to a slight decrease in rank. In line with the findings for the five focus countries, the differences across health-needs frameworks within each country category were quite small.

#### Broader comparisons

Compared with the health-needs frameworks, there were more variation within the capacity frameworks, within the impact frameworks, and within the other two-criterion frameworks in how the different frameworks affected different countries and country categories. As shown in Table 4, the Spearman correlation coefficients between GNIpc and each of the capacity frameworks ranged from 0.73 (Debt) to 0.99 (GHEpc). For the impact frameworks, coefficients ranged from 0.90 (framework integrating absolute improvement in under-five mortality rate (rcU5MR)) to 0.98 (rcSBA), and for other two-criterion frameworks they ranged from 0.75 (Gini) to 0.93 (DTP3). The correlation coefficients for the multi-criterion frameworks were 0.97 (MCF_EQ) and 0.95 (MCF_SU).

**Figure 3.**
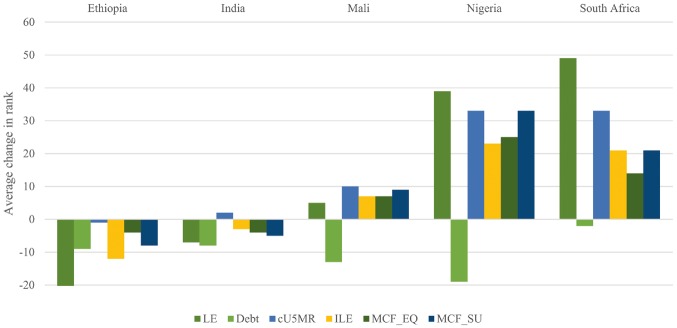
Changes in rank when moving from GNIpc alone to other frameworks (focus countries). LE, framework integrating life expectancy; Debt, framework integrating debt service; cU5MR, framework integrating absolute improvement in under-five mortality rate; ILE, framework integrating inequality in life expectancy; MCF_EQ, framework integrating life expectancy, inequality in life expectancy, and skilled birth attendance rate with use of equal weights; MCF_SU, framework integrating life expectancy, inequality in life expectancy, and skilled birth attendance rate with use of weights informed by online survey of stakeholders

As for the health-needs frameworks, even high correlation coefficients allow for substantial changes in rank for individual countries. Figure 3 exhibits how the ranks of the five focus countries changed as one moved from GNIpc alone to a broad set of other frameworks. This set includes health-needs, capacity, and impact frameworks, as well as the two multi-criterion frameworks. Changes associated with all frameworks and for all countries are provided in Supplementary File S2.

Nigeria and South Africa got a substantially higher rank when moving from GNIpc to any other framework, except for the debt framework. A similar but less marked pattern applied to Mali. Conversely, Ethiopia and India experienced a lower rank when moving to any other two-criterion framework, except the cU5MR framework for India. For the multi-criterion frameworks (MCF_EQ and MCF_SU), changes in rank were pronounced for Nigeria and South Africa. The difference between the multi-criterion framework with equal weights and that with survey-informed weights was modest.

**Figure 4.**
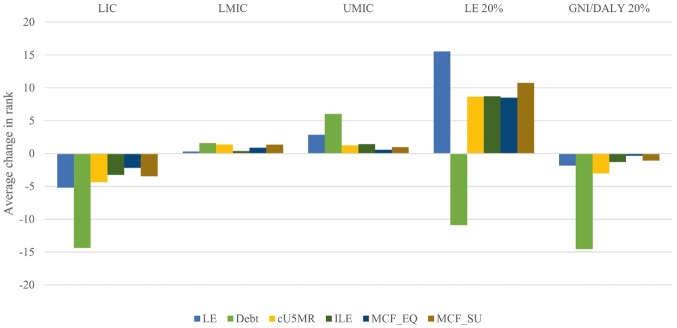
Average changes in rank for countries when moving from GNIpc alone to other frameworks (country categories). LE, framework integrating life expectancy; Debt, framework integrating debt service; cU5MR, framework integrating absolute improvement in under-five mortality rate; ILE, framework integrating inequality in life expectancy; MCF_EQ, framework integrating life expectancy, inequality in life expectancy, and skilled birth attendance rate with use of equal weights; MCF_SU, framework integrating life expectancy, inequality in life expectancy, and skilled birth attendance rate with use of weights informed by online survey of stakeholders; LIC, low-income countries; LMIC, lower-middle-income countries; UMIC, upper-middle-income countries; LE 20%, 20% of countries with the lowest life expectancy; GNI/DALYR 20%, 20% of countries with lowest ratio of GNIpc to DALYR

With regard to the country categories, Figure 4 shows the average changes in country rank as one moved from GNIpc alone to other frameworks. Changes associated with all frameworks and for all country categories are provided in Supplementary File S3.

This figure demonstrates some general trends. Among the income classes, all moves beyond GNIpc alone led to a lower rank on average for LICs and a slightly higher rank for LMICs and UMICs as groups. Among the two other categories, the 20% of countries with the lowest life expectancies got a higher rank, with the exception of the debt framework, whereas the countries with the lowest GNI-DALY ratios consistently got a lower rank. These patterns of change apply for the most part also to the frameworks not shown in Figure 4 (see Supplementary File S3). The exceptions are the GHEpc and Gini frameworks for the LMIC category, and the rcU5MR, cSBA, and rcSBA, SBA, and OOPP frameworks for the category of countries with the lowest life expectancy. In general, nearly any move beyond GNIpc alone that was examined in this study resulted in a lower rank for low-income countries as a group.

## Discussion

The findings from this study can inform donors in the assessment and reform of their allocation policies, as well as stakeholders and researchers contributing to such efforts. Our findings suggest that health needs, domestic capacity, expected impact and equity are all concerns that most stakeholders find relevant and that many of these stakeholders rank ‘needs and health needs’ as the top concern. The findings further suggest that nearly any departure from GNIpc alone will disfavour LICs as a group. While revision of a classification framework needs not be a zero-sum game, this finding serves as a reminder that any effort to target MICs should be accompanied by a close look on the consequences for LICs. The stagnation of DAH in recent years only underscores the importance of a careful examination.

### Health needs

Our findings suggest that addressing health needs is generally considered a key objective for DAH and that a general health-needs indicator can be relevant for many institutions. The findings further suggest that the indicators integrated in the six health-needs frameworks (U5MR, U60MR, LE, HALE, DALYR and DALYR_AS) all perform well in terms of relevance, salience, validity, consistency, and availability and timeliness. The comparison of frameworks indicates that including a health measure in the framework makes a substantial difference for many countries and country categories and that it improves the ranking and priority of countries with large unmet health needs in particular. Against this background, donors have reasons to combine GNIpc and one health-needs indicator for the initial classification of countries. If this is done, donors and other stakeholders may want to pay particular attention to any negative effects on LICs and provide safeguards where needed.

The findings also indicate that what specific health-needs indicator is used makes less of a difference than whether or not such an indicator is included at all. Moreover, no single health-needs indicator appears to be clearly superior to all others in terms of relevance, salience, validity, consistency, and availability and timeliness. However, our analysis suggests that both life expectancy and DALYR are good candidates for being included in a classification framework and that donors may want to pay particular attention to these if they want to use a single broad health-outcome measure. As described above, both life expectancy and DALYR are sensitive to all deaths, at all ages and from all causes. Life expectancy is already a familiar and well-tested measure in numerous contexts, including as one of three dimensions of the Human Development Index, where GNI per capita is another dimension (UNDP 2015). Moreover, life expectancy estimates are provided by several reliable institutions, including the UN Population Division, available for all countries and frequently updated. At the same time, while DALYR lacks some of the features of life expectancy, it exhibits direct sensitivity to morbidity and facilitates decomposition of health needs with regard to cause, risk factor and age structure.

### Other concerns

Findings from the literature review and the interviews suggest that domestic capacity, expected impact and equity are also important considerations when allocating DAH. However, none of the examined indicators was clearly suitable for inclusion in a classification framework when judged against the criteria of relevance, salience, validity, consistency, and availability and timeliness. While most the indicators were linked to general concerns that were found to be relevant, the relevance of the indicators specifically for use in a classification framework was less clear. A key reason is that the relationship between each of the indicators and rank in a classification framework appeared more convoluted than for the health-needs indicators. Many of the other indicators were also found to score relatively poorly on validity, consistency, or availability and timeliness.

Indicators other than the health-needs indicators can play an important role in the allocation of DAH even if the assessments above are accurate. First, these other indicators may be part of the classification framework used by donors with quite specific mandates. For example, the HIV prevalence rate may be key for a donor specifically focusing on HIV/AIDS. Second, one or more of the other indicators may be part of comprehensive multi-criterion frameworks, where the combination of indicators counteract perceived shortcomings of the individual indicators. Third, the other indicators may play a role at stages in the decision-making process different from the initial classification of countries. In particular, indicators of domestic capacity (other than GNIpc), expected impact and inequality may be considered as part of more discretionary assessments at the program or project level. These can be assessments in the review of applications or assessments during the implementation phase.

For indicators found to be relevant but poor performers in terms of validity, consistency, or availability and timeliness, donors and other stakeholders should identify the most relevant indicators and increase their efforts in the development of new metrics, collection of data or both.

### Limitations and future study

Some of the limitations to this study apply to most or all classification frameworks. One is that for nearly any normative view, it is impossible to combine all relevant concerns and indicators into one simple framework or index. Most or all frameworks also struggle to deal adequately with incentives and the problem that if poorer levels of an indicator imply higher priority for DAH, countries’ incentives to improve on this indicator is likely to be reduced. Similarly, most or all frameworks fail to deal adequately with pockets of poverty and unmet health needs. Here, one way forward could be to use health outcome data for the poorest quintiles, for example from Demographic and Health Surveys.

Pursuing research in the context of a process like the EAI comes with both strengths and weaknesses. One strength is that the research is oriented towards issues of direct interest to policy makers. One weakness is independence and the risk that the research leaves important but controversial issues unaddressed. In the present study, this was the case with regard to the study of explicit eligibility thresholds. However, while some of the limitations of our approach were the result of restrictions in the mandate given by the EAI, most were the result of deliberate trade-offs in the face of time constraints.

First, we focused on frameworks relevant to decisions on eligibility for financial assistance and the size of allocations and frameworks meant to apply early in the decision-making process. Future studies should examine normative bases and frameworks for decisions on the modality of assistance, non-financial support and the financing of global public goods for health. In addition, for decisions on modality and non-financial support, the development of robust inequality measures may be particularly important.

Second, the interviews and survey were based on a limited and non-representative sample of stakeholders. There were also considerable variation around several of the typical responses. While the interviews do not provide the ‘true’ answers for what donors *should* do, findings from the interviews can provide useful input to donors’ own deliberations. Future studies may explore concerns and indicators in greater depth and involve larger and more representative samples of stakeholders. Another article in this series reports on the online survey described above, which involved a larger number of stakeholders and examined the relative importance assigned to different indicators in greater depth than in the present study (cross-reference to article by Grépin *et al.* in this issue).

Third, we used GNIpc as the starting point and a component of all frameworks. This does not preclude putting less weight on GNIpc than other indicators in the frameworks, but our approach is less relevant for those who would like to entirely exclude GNIpc.

Fourth, we could only conduct a basic assessment of the many indicators’ salience, validity, consistency, and availability and timeliness. A valuable next step would be a fuller, in-depth assessment of the quality of the key candidate indicators for guiding the allocation of DAH. Such an exercise could be modelled on previous endeavours in neighbouring areas, such as the recent assessment of indicators by the Primary Health Care Performance Initiative (PHCPI 2015).

Fifth, we were unable to examine all relevant indicators, all relevant combinations of indicators and all plausible prioritization rules. We believe, however, that the indicators and frameworks examined in this study cover an informative range of alternatives and that the stepwise approach developed can be useful to donors also independently of the specific indicators and frameworks addressed here. Given the importance of generic health-needs indicators, future studies may critically examine the strengths and weaknesses of these indicators. Using assessments of two-criterion frameworks as the starting point, future studies may also explore more complex options. This may include examination of how well-established multi-criterion frameworks, such as the allocation formula used by the International Development Association and the criteria set used to identify Least Developed Countries, can better integrate a concern for unmet health needs.

Sixth, there is no agreement on the best method for framework or index construction, including on the best methods for normalization, weighting and aggregation. For example, while there may be widely agreed that health need, domestic capacity and expected impact are relevant concerns, there will be reasonable disagreement about their exact relative weights. At the same time, we found that the difference between the multi-criterion framework with equal weights and that with survey-informed weights was modest. More generally, while many methodological choices were somewhat arbitrary, we used simple and conventional methods throughout, with the aim of making the method as intuitive and transparent as possible. Future studies may examine the desirability and implications of different methodological choices in the specific context of DAH, including a comprehensive sensitivity analysis of these choices.

Seventh, we examined implications mainly in terms of changes in rankings. This only provides an indication of the implications for most decisions on DAH. The final classification of countries depends not only on the dimensions in which countries are assessed but also on the thresholds applied within those dimensions. These thresholds may or may not link directly to decisions regarding eligibility and transition. Among the eligible countries, the levels of funding are likely to be guided not primarily by country rank, but by cardinal scores and absolute differences in the relevant indicators or indices. Future studies should thus go beyond the pure ranking of countries to examine cardinal scores, absolute differences and the implications of different thresholds.

## Conclusion

The complex landscape for DAH calls for carefully crafted allocation policies that go beyond GNIpc. The findings of this study can inform donors in assessing and reforming their policies. Specifically, donors have reasons to include one health-needs indicator in the initial classification of countries, and both life expectancy and DALYR are indicators worth considering. For most donors, the findings further suggest that indicators related to other concerns may be mainly relevant at different stages of the decision-making process, require better data or both.

## Supplementary Data

Supplementary data are available at *HEAPOL* online.

## Ethical approval

For the discrete choice experiment, an IRB human subjects exemption status was obtained from New York University.

## Supplementary Material

Supplementary File 1

Supplementary File 2

Supplementary File 3
